# Analysis of the effect of dual reference lines on first positioning accuracy in intensity‐modulated radiotherapy for cervical cancers

**DOI:** 10.1002/acm2.70342

**Published:** 2025-11-23

**Authors:** Jinjin Feng, Zaichun Shang, Binbin Ge, Kaiyue Chu, Jianhua Jin, Jingjing Shao, Xinhua Zhang

**Affiliations:** ^1^ Department of Anatomy Medical School of Nantong University Nantong China; ^2^ Department of Radiotherapy Nantong Tumor Hospital Tumor Hospital Affiliated to Nantong University Nantong China; ^3^ School of Electronic Science and Engineering Nanjing University Nanjing China

**Keywords:** cervical cancer, dual positioning reference line, modified planning target volume, positioning error, radiotherapy

## Abstract

**Objective:**

To investigate the use of dual‐reference lines to reduce positioning errors and optimize modified planning target volume (MPTV) in volume modulated arc therapy (VMAT) for cervical cancers.

**Methods:**

Thirty‐seven patients with FIGO stage IIB‐IVA cervical cancer and no distant metastasis, who underwent radiotherapy in a tertiary hospital from June 15 2022 to September 15 2023, were selected and randomly divided into dual positioning reference line group (dual‐line group, 21 cases) and single positioning reference line group (single‐line group, 16 cases). A single reference line was made in pelvic region in single‐line group, while dual reference lines were made in stable abdominopelvic region in dual‐line group. The cone‐beam computed tomography (CBCT) was conducted to determine the positioning errors and calculate MPTV.

**Results:**

Linear error in *Y* direction, rotational errors in the rotation around *Y*‐axis (*RY*)/*Z*‐axis (*RZ*) (0.27 ± 0.12 cm, 0.60 ± 0.42°, 0.48 ± 0.44°) in dual‐line group were smaller than those (0.35 ± 0.22 cm, 0.78 ± 0.45°, 0.85 ± 0.66°) in single‐line group (*p* < 0.05). The thresholds of 0.4 cm and 1.4° were set as the boundary values for linear and rotational errors, respectively. There were significant statistical differences in the distribution of positioning errors in the six directions between the two groups (*p* < 0.001), with higher positioning error rates in the *Y* (77.78%), *RY* (41.46%), and *RZ* (53.66%) directions, respectively. Median total positioning time in dual‐line group (8.27 min, interquartile rang [IQR]: 7.65–8.63) was shorter than that (8.75 min, IQR: 7.89–9.45) in single‐line group (Z = 3.53998, *p *< 0.001). MPTVs in *X*, *Y*, and *Z* directions (0.25, 0.37, and 0.10 cm) in dual‐line group were smaller than those (0.31, 0.56, and 0.11 cm) in single‐line group.

**Conclusion:**

Dual‐reference lines improve positioning accuracy, reduce MPTV, and enhance efficiency in VMAT for middle and advanced cervical cancers, offering a clinically practical solution for precision radiotherapy.

## 1 INTRODUCTION

Cervical cancer is a common type of gynecological tumor, especially in developing countries, where it accounts for a significant percentage of morbidity and mortality in women with malignant tumors.^1^ For patients with early‐stage cervical cancers, radical radiotherapy has been proven to be able to achieve the effect of radical surgery; radiotherapy combined with chemotherapy is currently the standard method for the treatment of patients with middle and late‐stage cervical cancers.^2^, ^3^, ^4^ Radiotherapy techniques have evolved from two‐dimensional to three‐dimensional approaches such as intensity‐modulated radiotherapy (IMRT) and volumetric modulated arc therapy (VMAT), etc.^5^ Among these techniques, VMAT has become an important technical means in external irradiation for cervical cancer due to its advantages such as high conformability, less damage to adjacent organs, and shorter treatment time.^6^ Although, the VMAT technique offers significant therapeutic advantages, it imposes higher requirements on patient positioning reproducibility during treatment. However, in cervical cancer radiotherapy, the positioning error is a key factor affecting radiotherapy accuracy, with a significant effect on the irradiated doses in modified planning target volume (MPTV) and organs at‐risk (OARs). Tsujii et al. reported that the positioning error is moderately correlated with the clinical tumor volume (CTV) coverage (*r* ≥ 0.40) and strongly correlated with the volume percentage of the bladder exposed to 45GY irradiation (V45Gy, *r* ≥ 0.91) and the volume percentages of rectum, small intestine, bone marrow exposed to 40GY irradiation (V40Gy, *r* ≥ 0.91).^7^ Khan et al. analyzed inter‐fractional positioning errors in 50 cervical cancer patients, and found that to achieve 95% CTV coverage, the planning target volume (PTV) needed to be uniformly expanded in *X*,*Y*, and Z directions by 13 mm or anisotropically expanded by 10, 10, and 20 mm, respectively.^8^ At present, surface‐guided radiotherapy (SGRT) is being gradually applied for tumors in the head and neck, chest, and abdomen. The study by Rudat V. et al. revealed that compared with traditional laser‐guided positioning, the surface‐guided positioning can reduce the translational positioning error by 0.9 mm in tumor SGRT.^9^ Traditional single reference line technique relies on a single anatomical landmark. This approach may compromise the reproducibility of inter‐fractional positioning of the long target volume in the pelvic cavity due to skin deformation or involuntary movements in patients. However, the surface‐guided positioning cannot fully compensate for such systematic errors^10^ and high‐precision positioning still requires reliable body surface markers. The application of the surface‐guided positioning as an auxiliary tool in cervical cancer radiotherapy can improve positioning efficiency and correct intra‐fraction deviations.^11^ The cone‐beam computed tomography (CBCT) currently remains the conventional method used by most radiotherapy centers to validate intra‐fraction and inter‐fraction positioning errors. The study by Stanley et al. showed that the overall 3D shift correction at different sites in patients initially aligned using a surface imaging system is significantly smaller than that in patients aligned using reference lines^12^ but the body surface reference line method still has application value in tumor radiotherapy. It is not advocated to completely remove the reference line‐based alignment method. A study by Lee et al. showed that there is no statistically significant difference in final CBCT shift values between surface and skin marker guided positionings in IMRT for prostate cancers.^13^

In radiotherapy for middle and advanced cervical cancer, the CTV includes the primary lesion, the drainage area of the abdominal para‐aortic lymph nodes, and the drainage area of the pelvic lymph nodes. The longitudinal extent is usually larger. Consequently, the positioning based on a single reference line may not be reliable enough. Secondly, due to the special anatomical structure of cervical cancer, the positioning accuracy is easily affected by factors such as radiotherapy position, fixation mode, bladder filling, body mass index (BMI), and skin pulling.^14^ Meanwhile, there are fewer reports on the reproducibility of positioning for cervical cancer radiotherapy. Therefore, in order to improve the positioning reproducibility and treatment accuracy of radiotherapy for cervical cancer, it is crucial to select a suitable area to delineate the body surface positioning reference lines. In this study, the CBCT was used, dual positioning reference lines were made in the relatively stable abdominopelvic region, and the effect of dual positioning reference lines in radiotherapy for cervical cancers was investigated. This study aims to evaluate the potential effect of dual positioning reference lines in reducing the positioning errors and rotational errors and further investigate its effect on MPTV in the treatment plan. We hope this study can provide the cervical cancer patients with a simple, feasible, and highly accurate positioning technology, thereby improving treatment effectiveness and reducing unnecessary side effects.

## 2 METHOD

### 2.1 Case selection and general information

Thirty‐seven patients with cervical cancers who underwent VMAT in a tertiary hospital from June 15 2022 to September 15 2023 were enrolled into this study. The patients aged 33–83 years old, with a median age of 59 years old. They were pathologically diagnosed with FIGO stage IIB‐IVA cervical squamous carcinoma, without distant metastasis. Their Karnofsky Performance Status (KPS) scores were greater than 90 points. All patients were divided into the dual positioning reference line group (dual‐line group, 21 cases) and the single positioning reference line group (single‐line group, 16 cases) according to the random number table. All patients had postoperative pathological diagnosis; none of them had contraindications to radiotherapy. As shown in Table 1, there were no statistically significant differences in general information between the two groups (*p *> 0.05), indicating that the two groups were comparable. This study involving all subjects was reviewed and approved by the ethics committee of our center (approval number:2022–03).

**TABLE 1:** The characteristics of patients.

	Single‐line group	Dual‐line group	*p*
No of. patients	21	16	
Age (years), mean ± SD	58.48 ± 12.03	59.19 ± 11.82	0.859
Baseline weight (kg), mean ± SD	59.38 ± 9.43	61.31 ± 9.97	0.551
BMI (kg/m^2^), mean ± SD	24.80 ± 4.49	25.16 ± 4.83	0.813
Hysterectomy, *n* (%)			
Yes	12(57.14)	9(56)	
No	9(42.86)	7(44)	
FIGO stage, *n* (%)			
IIB‐IIIA	14(66.66)	10(62.50)	
IIIB‐IVA	7(33.34)	6(37.50)	

SD, standard deviation.

### 2.2 Position fixation and reference line delineation

Patients in both groups were immobilized using a vacuum mold in combination with a thermoplastic film. At the 1 h before the molding procedure, the patient needed to undergo bowel preparation, empty the bladder, and drink 500 mL of water, and hold urine. When the patient felt the urge to urinate, a bladder scanner was used to measure bladder volume, and the urine volume and the duration of holding urine were recorded. During the molding procedure, the vacuum molding was performed according to the body contours of the patient, and the vacuum mold was secured to an integrated immobilization board using fixtures. The patient was asked to raise both arms to grasp the handgrips of the board while lying supine on the vacuum mold. Two W‐shaped feet supports were then placed under both feet to ensure a stable and comfortable position. The patient should breathe calmly, with full relaxation. The waist and perineum of the patient should be immobilized using the vacuum molds, with both legs slightly separated to ensure proper fit. After vacuum molding, reference lines were made under the guidance of a laser light.

**FIGURE 1:**
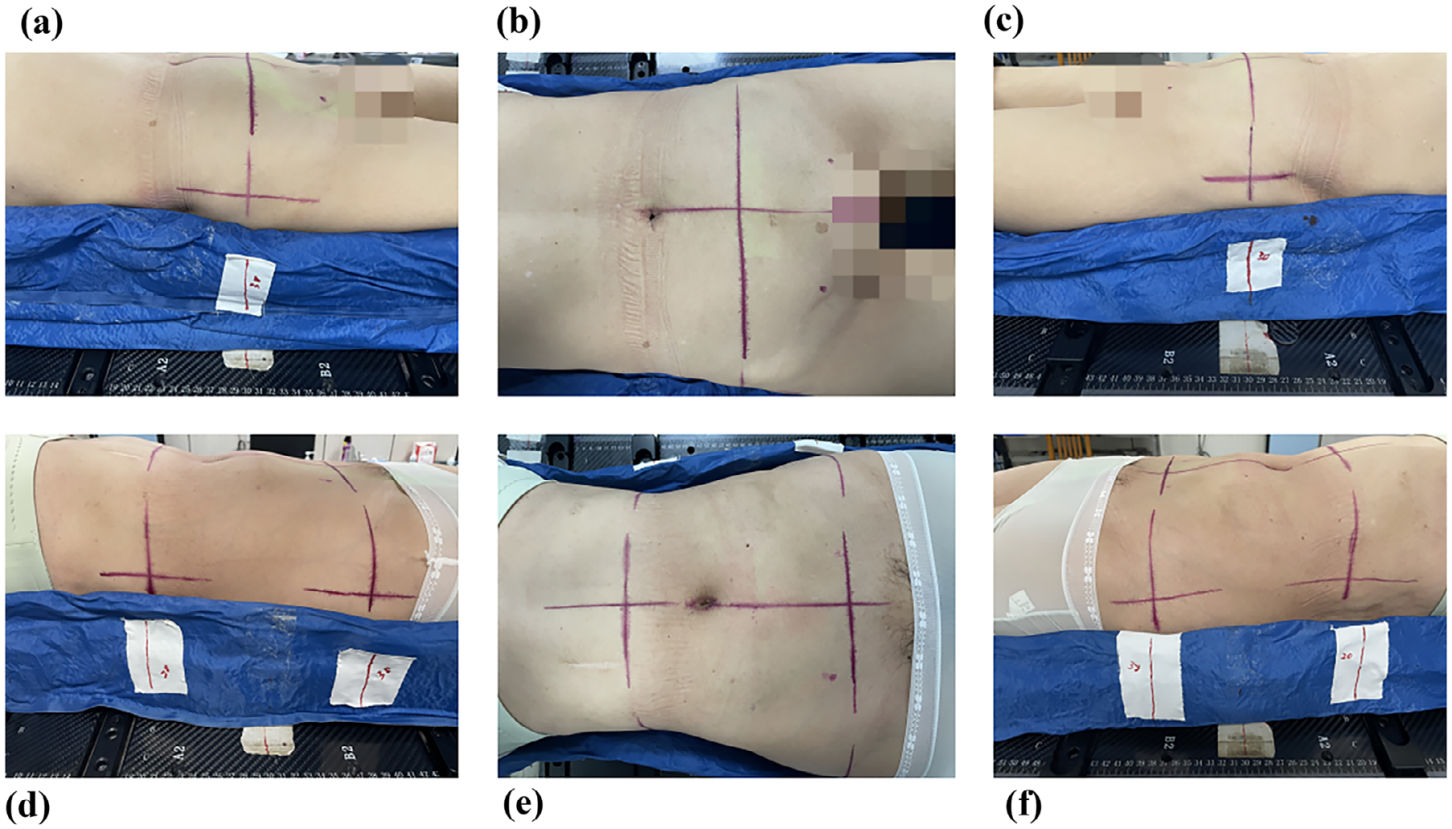
Positioning reference lines in two groups. (a), (b), and (c): single positioning reference line in a patient from the single line group; (d), (e), and (f): dual positioning reference lines in a patient from the dual line group.

**Single line group**: The transverse axis reference line was drawn in the abdomen and both sides at 1 cm from the lower margin of the iliac spine. A reference line was drawn at the corresponding position on the vacuum mold, and the corresponding scale value in the integrated immobilization board was recorded. The midaxillary line and the body midline were taken as the baselines to draw the coronal and sagittal axis reference lines respectively on skin surface (see Figure 1a–c).

**Dual line group**: The upper positioning reference line was drawn at the lower margin of the costal arch, and the transverse axis reference line on the upper abdomen and both sides were drawn on the skin surface; the treatment couch was moved by a fixed distance (recorded) toward the connection line between bilateral acetabulums (about 3 cm above the pubic symphysis), where the lower positioning reference line was delineated, and the transverse axis reference line on the lower abdomen and both sides were drawn on the body skin surface, the reference line was made on the corresponding position in the vacuum mold, and the scale value in the integrated immobilization board was recorded. The midaxillary line and the body midline were taken as the baselines to draw the two sets of coronal and sagittal axis reference lines on the upper and lower skin surface (see Figure 1d–f).

The reference lines were drawn using medical erasable ink, a method used in our center for many years. The ink was highly safe, caused little skin irritation, and could be redrawn under laser guidance if blurred by sweat or friction, thus avoiding patient anxiety caused by permanent markings.

After the reference lines were drawn, a heated and softened thermoplastic film was applied to cover the body surface and further shaped. Finally, the buckles at both ends of the thermoplastic film were fastened to complete the fixation.

### 2.3 CT positioning and target volume delineation

CT (Brilliance TM, Philips, Amsterdam Holland) was performed on the second day, and the patients in two groups underwent repeated bowel preparation during molding before CT scanning, and the bladder volume was monitored using a bladder scanner, and if the deviation value between the current bladder volume of the patient and that during molding exceeded 20%, the patient was instructed to pass a small amount of urine or prolong the time of holding urine until the deviation value was less or equal to 20%, and the urine volume and the time of holding urine were recorded.^15^ The patient lay on the vacuum mold, and the thermoplastic film was immobilized after the patient's position was adjusted to make two sets of positioning reference lines, which completely coincided with the reference lines in the vacuum mold and the scale line in the integrated immobilization board under the guidance of LAP laser light system. The therapist used the LAP laser light system to draw three cross‐reference lines above and on both sides of the thermoplastic film according to the irradiation range specified by the clinician, and affixed metal markers to determine the isocentric coordinate. The patient was scanned using a Philips large‐aperture CT simulator, and the scanning range was from the bifurcation of the abdominal aorta to the lower margin of the obturator foramen, with a layer thickness of 3–5 mm. The patient underwent arteriography to clearly visualize the tumor and blood vessels. The positioning CT images were transmitted to the treatment planning system (TPS), (Pinnacle3 9.0, Philips, Holland), and the target volume was delineated by an experienced radiation oncologist according to the target delineation principles in the International Commission on Radiation Units and Measurements (ICRU) Report No. 62 in combination with relevant literature. The gross tumor volume (GTV) included the cervical tumor and its invasion area, enlarged pelvic lymph nodes, and enlarged lymph nodes around the abdominal aorta, while the CTV included the cervix, uterus, para‐uterine tissues, and pelvic lymph node regions.^16^, ^17^

### 2.4 Treatment positioning and data collection

The first radiotherapy was performed together by the radiation oncologist, physicist and radiotherapist to ensure accuracy and consistency. During positioning, the patient underwent repeated bowel preparation and position fixation. In the dual line group, the couch was displaced to the predetermined distance following alignment of the upper pendulum reference line. The patient was gently moved to align the lower positioning line. Subsequently, the upper positioning line was rechecked. Then, the patient was covered with thermoplastic film. The treatment couch was moved to align the laser line to the radiotherapy isocenter. The x‐ray volumetric imaging (XVI) (Synergy, Elekta, Sweden) was used by an experienced therapist to perform CBCT to obtain images, which were then matched with the positioning images from the planning system to assess the accuracy of the patient positioning. In the matching process, the automatic bone registration (translation + rotation, T+R) was first performed, then manual adjustments were made to optimize the bone registration results. The positioning errors after image registration were recorded, including the linear errors in left–right (*X*), cranio–caudal (*Y*), and antero–posterior *(Z*) directions, and the rotational errors in the rotation around the *X*‐axis (*RX*), the rotation around the *Y*‐axis (*RY*) and the rotation around the *Z*‐axis (*RZ*). CBCT was done three times during the first week of treatment and once weekly thereafter. The allowable error range was defined as a linear error ≤ 0.5 cm and a rotational error ≤ 3°. As our center had not been equipped with a six‐dimensional couch, any errors exceeding these limits required repositioning and correction before treatment.

### 2.5 Evaluation indicator

#### 2.5.1 Positioning errors in the two groups

To compare the absolute values of the six‐dimensional directional errors between the two groups. The data with absolute linear errors less than 0.4 cm in all *X*, *Y*, and *Z* directions were divided into one group, while the data with absolute linear error values greater than or equal to 0.4 cm in any direction were divided into another group. The data with rotational errors less than or equal to 1.4° in the *X*, *Y*, and *Z* directions were divided into one group, while the data with rotational errors greater than 1.4° in *X*, *Y*, and *Z* directions were divided into another group. The overall positioning error and the distribution of positioning errors in each direction were compared between the two groups.

#### 2.5.2 Positioning time

Positioning time was defined as the total time required to achieve clinically acceptable positioning accuracy, including the placement of a patient on the treatment couch, position adjustment, bladder monitoring, alignment of surface markings with laser lights, immobilization using a thermoplastic film, CBCT image acquisition, image registration, and verification. The positioning time corresponding to all planned CBCT acquisitions in both groups was recorded, and interruption time caused by non‐operational factors such as sudden patient discomfort or equipment malfunctions was excluded. A built‐in timer in the radiotherapy management system was used to record the time, which was accurate to the 0.1 min.

#### 2.5.3 Calculation of MPTV value

To ensure that 90% of CTV received at least 95% of the prescribed radiation dose, the MPTV from the CTV to the PTV needed to be calculated. The following formula was used:^18^

(1) $$\mu_{ptv}=2.5\Sigma +0.7\delta$$

In the formula, Σ was the standard deviation of the systematic error, which was the standard deviation of the mean value of the patient positioning errors. *δ* was the standard deviation of random error, which was the root‐mean‐square deviation of the patient positioning errors.

### 2.6 Statistical methods

In this study, SPSS 24.0 and Origin Pro 9.6.5 software were used for statistically analyzing and graphing the collected data. Firstly, Kolmogorov–Smirnov (K–S) test was performed on the absolute values of the measurement data (positioning errors). The data conforming to normal distribution were compared between the two groups using the independent samples *t*‐test and expressed as mean ± standard deviation. The data not conforming to normal distribution were compared between the two groups using the Wilcoxon rank sum test. The data distribution was expressed as frequency (*n*) and percentage (%), and the Chi‐square test was used to compare the distribution of linear errors between two groups. The median (interquartile range, IQR) was used to describe the centralized tendency and variation degree during the six positioning times in each group. Intergroup comparisons were performed using the Mann–Whitney *U* test. The significance level was set at *α* = 0.05 (two‐tailed), and *p* < 0.05 was considered statistically significant.

## 3 RESULTS

### 3.1 Comparison of linear and rotational errors between two groups

**FIGURE 2:**
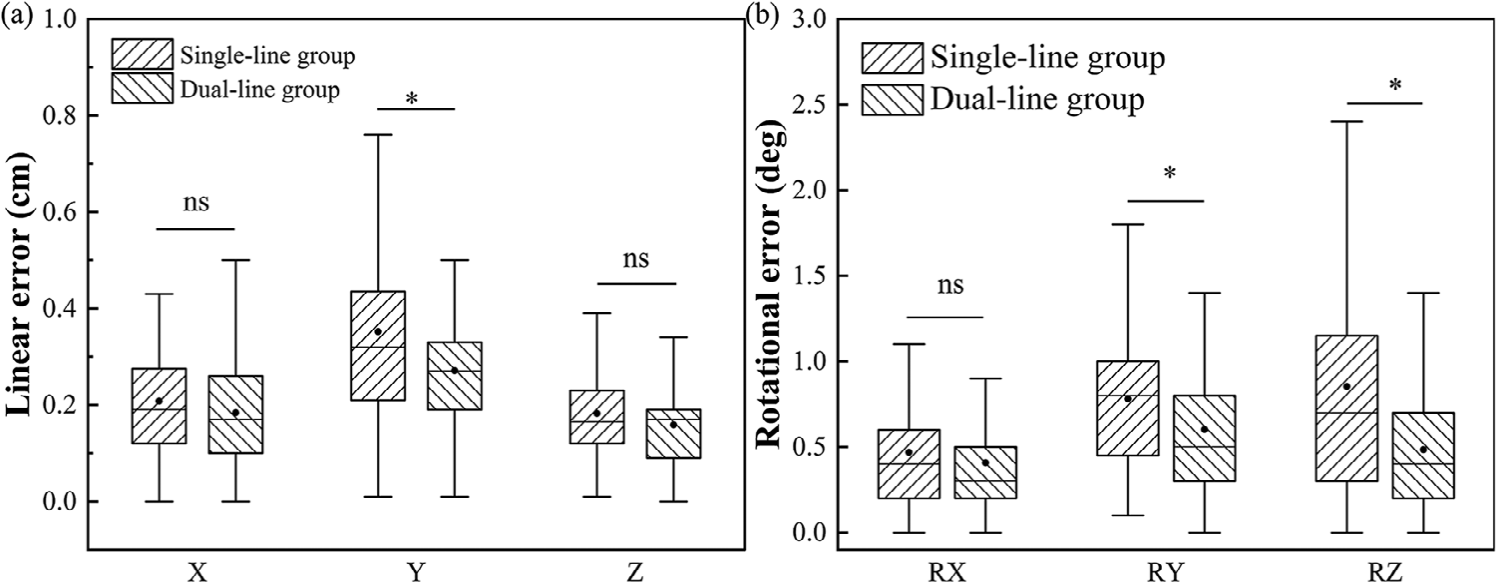
Box plots for the comparison of six‐dimensional directional patient positioning errors between two groups. (a) comparison of linear errors in *X*, *Y*, and *Z* directions between; (b) comparison of rotational errors in *RX*, *RY*, and *RZ* directions between two groups. Note: * represents *p* < 0.05, ns represents no statistical significance, and ● in the box plots represents average value.

A total of 267 sets of CBCT scanning data were collected from 37 patients. The data were tested for normality distribution and showed a skewed normal distribution. As shown in Table 2, the linear error in the *Y*‐direction in the dual‐line group was significantly smaller than that in the single‐line group, with a statistically significant difference between two groups (*p *< 0.05, *t *= 3.644). As shown in Table 3, the rotational errors in the *RY* and *RZ* in the dual‐line group were significantly smaller than those in the single‐line group, with statistically significant differences between two groups (*p *< 0.05, *t *= 3.388; *p *< 0.05, *t *= 5.261). The comparison of the positioning errors in six dimensions between the two groups was shown in Figure 2, indicating that the application of dual positioning reference lines effectively reduced the linear error in *Y*‐axis direction and the rotational errors in *RY* and *RZ*, thus improving the precision of the radiotherapy.

**TABLE 2:** Comparison of linear errors in *X*, *Y*, and *Z* directions between the two groups (cm).

Group	*n*	*X*	*Y*	*Z*
Single‐line group	120	0.21 ± 0.13	0.35 ± 0.22	0.18 ± 0.11
Dual‐line group	147	0.18 ± 0.12	0.27 ± 0.12	0.16 ± 0.10
*t*		1.557	3.644	1.810
*P*		0.121	<0.05	0.071

**TABLE 3:** Comparison of rotational errors in *RX*, *RY*, and *RZ* between the two groups (deg).

Group	*n*	R*X*	*RY*	*RZ*
Single‐line group	120	0.47 ± 0.37	0.78 ± 0.45	0.85 ± 0.66
Dual‐line group	147	0.41 ± 0.32	0.60 ± 0.42	0.48 ± 0.44
*t*		1.424	3.388	5.261
*p*		0.156	<0.05	<0.05

### 3.2 Comparison of the distribution of patient linear errors between the two groups

**FIGURE 3:**
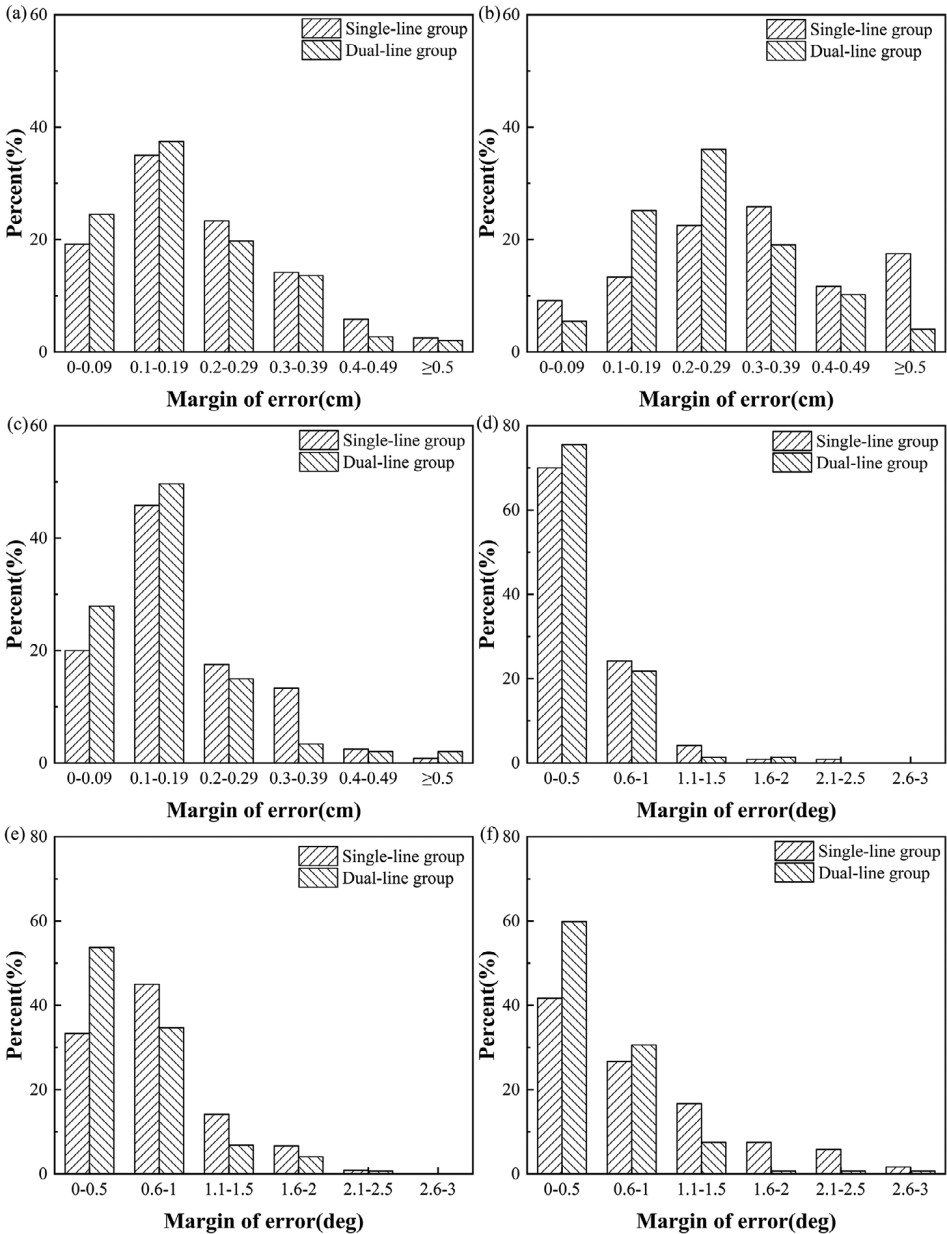
Distribution of positioning errors in six‐dimensional directions in patients of the two groups. (a), (b), and (c) show the distribution of linear errors in *X*, *Y*, and *Z* directions in two groups respectively; (d), (e), and (f) show the distribution of rotational errors in *RX*, *RY*, and *RZ* in two groups, respectively.

The distributions of three dimensionals directional (*X*, *Y*, and *Z*) patient linear errors in two groups were shown in Figure 3; it could be seen that compared with the single‐line group, the overall distribution of the linear errors in the dual‐line group was closer to 0 and more concentrated between 0 and 0.29 cm, with a lower percentage of greater error values.

Table 4 showed that compared with the single‐line group, a better positioning (absolute linear error < 0.4 cm) was more likely to be achieved in the dual‐line group; the percentage of absolute linear error < 0.4 cm in all directions was 80.27%, which was higher than 64.17% in the single‐line group, with a statistically significant difference between two groups (*χ* ^2^ = 8.701, *p *= 0.003).

**TABLE 4:** Comparison of linear error rate between the two groups with an absolute error of 0.4 cm as the cutoff value.

		Positioning error rate (%)		
		<0.4 cm	≥0.4 cm	Total
Group	Single‐line group	77(64.17)	43(35.83)	120
	Dual‐line group	118(80.27)	29(19.73)	147
Total		195(73.03)	72(26.97)	267
*χ* ^2^		8.701		
*p*		0.003		

An absolute linear error of 0.4 cm was taken as the cutoff value to compare linear positioning errors among *X*, *Y*, and *Z* directions. Table 5 showed that there was a statistically significant difference in the percentage of poor positioning (absolute linear error ≥ 0.4 cm) among three different directions (*χ* ^2^ = 74.582, *p* < 0.001), of which the percentage of poor positioning in Y direction reached 77.78%.

**TABLE 5:** Comparison of linear error rate among three different directions with an absolute error of 0.4 cm as the cutoff value.

		Positioning error rate (%)		
		<0.4 cm	≥0.4 cm	Total
	X	55(76.39)	17(23.61)	72
Positioning errors in three directions	Y	16(22.22)	56(77.78)	72
	Z	63(87.50)	9(12.50)	72
Total		134(62.04)	82(37.96)	216
*χ* ^2^		74.582		
*p*		0.000		

### 3.3 Comparison of rotational error distribution between the two groups

Figure 3 shows that the rotational errors in the dual‐line group were concentrated in the range of 0°–0.5°, with a lower percentage of larger errors compared with the single‐line group. Table 6 showed that the percentage of rotation errors ≤ 1.4° in all directions in the dual‐line group was 91.16%, which was significantly higher than 74.17% in the single‐line group, with a significant statistical difference between the two groups (*χ* ^2^ = 13.856, *p *< 0.001).

**TABLE 6:** Comparison of rotational error rate between the two groups with an absolute error of 1.4° as the cutoff value.

		Positioning error rate (%)		
		≤ 1.4°	> 1.4°	Total
Group	Single‐line group	89(74.17)	31(25.83)	120
	Dual‐line group	134(91.16)	13(8.84)	147
Total		223(83.52)	44(16.48)	267
*χ* ^2^		13.856		
*p*		0.000		

1.4° was used as the cutoff value, the quality of positioning errors was statistically analyzed and compared among the three directions (*RX*, *RY*, and *RZ*). As shown in Table 7, there was a significant statistical difference in the positioning error rate among three directions (χ^2^ = 16.207, *p* < 0.001); the positioning error rates in *RY* and *RZ* were 41.46% and 53.66%, respectively.

**TABLE 7:** Comparison of rotational error rate among three different directions with an absolute error of 1.4° as the cutoff value.

		Positioning error rate (%)		
		≤ 1.4°	> 1.4°	Total
	*RX*	36(87.80)	5(12.20)	41
Positioning errors in three directions	*RY*	24(58.54)	17(41.46)	41
	*RZ*	19(46.34)	22(53.66)	41
Total		79(64.23)	44(35.77)	123
*χ* ^2^		16.207		
*p*		0.000		

### 3.4 Comparison of the positioning time between the two groups

The median positioning time for six CBCT acquisitions in the dual‐line group was 8.27 min (IQR: 7.65–8.63), which was significantly shorter than 8.75 min (IQR: 7.89–9.45) in the single‐line group (*Z* = 3.539, *p *< 0.001). The time trend analysis showed that the first positioning time was the longest in both groups, with a median positioning time of 8.64 min in the dual‐line group and 8.92 min in the single‐line group. The third positioning time was shorter in both groups, but the dual‐line group always maintained an efficiency advantage over the single‐line group (see Table 8).

**TABLE 8:** Comparison of the positioning time between the two groups (min).

	Positioning time		
	Total positioning time	First positioning time	Third positioning time
Single‐line group	8.75(7.89–9.45)	8.93(8.37–10.43)	8.21(7.85–8.83)
Dual‐line group	8.27(7.65–8.63)	8.64(8.49–8.88)	7.64(7.27–7.84)
*Z*	3.539	1.533	3.974
*p*	<0.001	0.125	<0.001

### 3.5 Modified planning target volume (extended margin of target volume)

It was calculated according to Formula 1 that MPTVs in the *X*, *Y*, and *Z* directions were 0.31, 0.56, and 0.11 cm, respectively, in the single‐line group, and 0.25, 0.37, and 0.10 cm, respectively, in the dual‐line group (see Table 9), and MPTVs in three directions in the dual‐line group decreased by 19.4%, 33.9%, and 9.1%, respectively, compared with those in the single‐line group.

**TABLE 9:** Comparison of MPTV between the two groups (cm).

	Statistical value	*X*	*Y*	*Z*
Single‐line group	Σ	0.06	0.11	0.01
	σ	0.24	0.40	0.02
	MPTV	0.31	0.56	0.11
Dual‐line group	Σ	0.05	0.03	0.02
	σ	0.20	0.40	0.06
	MPTV	0.25	0.37	0.10

## 4 DISCUSSION

As a common type of gynecological tumor, the precision and positioning reproducibility of radiotherapy are important for the therapeutic effect of cervical cancers. Yao et al. emphasized that both linear and rotational errors must be addressed during radiotherapy for cervical cancers.^19^ Failure to correct these errors may result in underdosing of tumors and positive lymph nodes, potentially leading to tumor recurrence. ICRU Report 24 stated that a 5% dose deviation has the potential to cause uncontrolled tumors and/or increased complications in normal tissue.^20^ Therefore, it is particularly important to strengthen the control of positioning errors in cervical cancer radiotherapy. CBCT images can accurately and clearly display 3D structural changes in the tumor and surrounding normal tissues and organs in the treatment orientation. CBCT has the advantages of clear imaging, convenient operation and high degree of automation, and it can be used to easily obtain clearer positioning errors compared with the traditional electronic portal imaging device (EPID). CBCT is of great significance in precise radiotherapy for tumors.^21^, ^22^ This study compared the application effects of dual positioning reference lines and single positioning reference lines in IMRT for cervical cancers, and explored the potential of dual positioning reference lines in reducing the linear errors and rotational errors, and further analyzed its effect on MPTV.

With the updating of image‐guided techniques and equipment, the reference line alignment cannot be completely replaced, although the reliance on traditional reference line alignment has been greatly reduced. During radiotherapy for patients with cervical cancers, a large deviation in positioning accuracy usually occurs at each radiotherapy session due to fewer bony markers, thicker subcutaneous fat, relatively greater mobility, more internal organs, and larger deformations in this abdominopelvic region. In CBCT image‐guided radiotherapy, body surface reference lines are commonly used in China to improve positioning accuracy. However, there are currently differences in the positions of reference lines and the way for marking the lines. Pan et al. investigated the application of two different fixation and marking methods in pelvic tumor radiotherapy, indicating that the positioning errors in the six‐dimensional directions differed from those in this study.^23^ The probable reason for this is that their study only used the vacuum mold for patient fixation and only made the reference line at the lower margin of the coastal arch. In contrast, the fixation method of thermoplastic film combined with vacuum mold used in this study had a pressurizing effect on the abdominopelvic region, thus reducing the effects of factors such as respiration and fat thickness on the accuracy of positioning. Stanley et al. retrospectively analyzed and compared the accuracy of positioning in initially treated patients between surface imaging positioning and three‐point positioning^12^ and found that the mean magnitude of error in 3D directions in the pelvis and lower limbs was 0.90 ± 0.4 cm in patients with conventional three‐point alignment. Archana et al. marked four reference points at the pubic symphysis,^24^ two horizontal sides, and the lower margin of the xiphoid process; although the areas of these reference points are similar to those in this study, the positions of the points in relation to the positions of lines are more susceptible to skin pulling. Management of the whole course of bladder filling and corresponding bowel preparation were performed throughout the entire process from positioning to radiotherapy in the cervical cancer patients in this study, which reduced the rotational and linear positioning errors caused by changes in abdominal pressure due to bladder filling and intestinal gas accumulation. Our previous study showed that if the highly reproducible bladder volume deviation is less than 20%, the positioning error will be reduced, and thus, it is easier to obtain an excellent positioning status (error deviation < 0.4 cm), especially in the *Y* direction.^15^ In this study, the linear errors in all 3D directions in the dual‐line group were smaller than those in the single‐line group; the linear error in the *Y* direction in the dual‐line group was significantly smaller than that in the single‐line group (*p *< 0.001). The reason for this was that two reference lines were made, respectively, at bilateral acetabulums and the lower margin of the costal arch; these two areas were relatively stable in the abdominopelvic region. There are statistically significant differences in rotational error between *RY* and *RZ*. It has been shown that the rotational error in RX is greater than that in *RY* and *RZ*.^25^, ^26^ The reasons for these kinds of differences in this study are analyzed as follows: 1) There is a difference in the fixation method, the tumor patients often have emotions such as fear and anxiety; at the beginning of the whole treatment, the patients are prone to bend their waist because of stress. In this study, the patients in both groups were immobilized with vacuum molds, which were made according to the characteristics of human physiological curves to effectively support the waist and relieve stress. Thus, the rotational error in R*X* was reduced to a certain extent; 2) The target volume is usually longer in patients with cervical cancers. In the dual‐line group, it was needed to align the upper and lower reference lines, the vacuum mold and the scale value in the integrated immobilization board during each positioning, which can make the patient position during each treatment basically consistent with that during positioning, thus effectively reducing the positioning errors in *RY* and *RZ*. In the single‐line group, it was only needed to align a single reference line during positioning; it was impossible to ensure the reproducibility of the treatment positions at both ends of the target volume; a small rotation in the center of the target volume might cause a larger rotational error in *Y* direction. In the dual‐line group, the upper and lower positioning reference lines were drawn respectively at the levels of the lower margin of the costal arch and the connection line between bilateral acetabulums. The application of dual positioning reference lines can better fix the position of the patient and reduce the positioning error due to changes in body position. In addition, the linear and rotational error distributions in the dual‐line group were more concentrated between 0 and 0.29 cm, and 0° and 0.5°, respectively, and the percentage of greater error values was lower, indicating that the dual positioning reference lines can improve the reproducibility and stability of the positioning. In this study, the MPTV in the X, Y, and Z directions in the dual‐line group were 0.25, 0.37, and 0.10 cm, respectively, which were 19.4%, 33.9%, and 9.1% lower than those in the single‐line group, respectively. This suggests that the application of dual‐position reference lines may have potential clinical value in improving radiotherapy accuracy while reducing MPTV, but the actual boundaries should be determined by combining the institutional specificity.

Lnu et al. used a built‐in imaging to assess positioning errors in cervical cancer radiotherapy, indicating that individual systematic errors in the *X*, *Y*, and *Z* directions were(−0.24–0.17) cm, (−0.36–0.29) cm, and (−0.15–0.19) cm, respectively; MPTV value was calculated to be 7 mm.^27^ LI et al. studied the effects of positioning errors on distribution of radiotherapy dose in cervical cancer and the margin from CTV to PTV (MPTV), and the results revealed that the absolute values of linear errors in the *X*, *Y*, and *Z* directions were (1.4 ± 1.0) mm, (2.3 ± 1.5) mm, and (1.9 ± 1.2) mm, respectively, and MPTV values in three directions were 4.4, 6.4, and 5.8 mm, respectively.^28^ As shown in Formula 1, MPTV value is primarily correlated with Σ (the standard deviation of linear error), which is the key factor determining the width of MPTV. The smaller the error is, the narrower the required MPTV is, which can significantly reduce the radiation dose in OARs such as the bladder, rectum and small intestine. Laursen et al. investigated the residual rotational positioning errors after daily CBCT image‐guided radiotherapy for locally advanced cervical cancer, revealing that when the positioning errors in *RX* (1.4 ± 0.04°), *RY* (0.9 ± 0.04°), and *RZ* (0.9 ± 0.06°) are converted into linear errors, a 5 mm MPTV is obtained.^29^ The study by Miao et al. revealed that when the rotational errors exceeds 1.4°, PTV coverage can decrease by > 17%.^30^ Based on the above study results and combined with our center's clinical practice experience, the percentages of linear errors in the two groups were analyzed using an absolute positioning error of 0.4 cm as a cutoff value. The results showed that the percentage of positioning errors < 0.4 cm in the dual‐line group was greater than that in the single‐line group, indicating that the total linear error in the dual‐line group is smaller than that in the single‐line group. The linear errors in three directions were analyzed, and the percentage of positioning errors ≥ 0.4 cm in the *Y* direction reached 77.78%, indicating that the linear error in Y direction is larger in both groups, this result is consistent with those of most studies.^31^, ^32^, ^33^ 1.4° was used as the cutoff value, the percentages of rotational errors in the two groups were analyzed. The results showed that the percentage of rotational errors ≤ 1.4° in the dual‐line group was greater than that in the single‐line group, indicating that the total rotational error in the dual‐line group is smaller than that in the single‐line group. An analysis of rotational errors in three directions revealed that the percentages of rotational errors in *RY* and *RZ* exceeding 1.4° were 41.46% and 53.66%, respectively, indicating that both groups had significant rotational errors in the *RY* and *RZ* directions. This suggests that the dual‐line group can better ensure positioning reproducibility between radiotherapy fractions and achieve a more optimal positioning state compared with the single‐line group.

In this study, the dual‐line group significantly outperformed the single‐line group in terms of positioning time corresponding to multiple CBCT acquisitions (median 8.27 vs. 8.75 min, *p* < 0.001). The reference lines in dual‐line group were made in the relatively stable abdominal pelvic region, meanwhile the laser‐assisted calibration was used, and thus the positioning time further decreased with increased operational proficiency. In contrast, the single reference line was made near the umbilicus, which was susceptible to factors such as bladder volume and abdominal fat, making it difficult to align the abdominal cross line with the cross lines on both sides of the body in the same plane, which increased the time required for positioning operations. This indicates that dual‐reference line technique can enhance the long‐term operational efficiency by standardizing the reference line alignment process. All patients included in this study underwent standardized bladder and bowel preparation, they were required to have a fluid‐filled bladder. Prolonged positioning time may lead to changes in patient position, and alterations in bladder filling status can also cause variations in target dose.^34^ The advantage of short positioning time of the dual positioning reference lines can reduce such risks.

This study has some limitations. First, the sample size of this study was relatively small, which might lead to selection bias. Larger‐scale studies are required in the future to confirm our findings and quantify the dosimetric impact on treatment plans. Second, the follow‐up period in this study was relatively short, and the effect of the dual positioning reference lines on the long‐term treatment outcome of cervical cancers was not assessed. In addition, this study did not consider other factors that might affect the positioning errors. For example, the nonpermanent skin ink was used in our center, and sweating and friction of clothing could cause blurring of reference lines; although the colour was deepened again under the guidance of the laser light, and the differences in the reference lines between the marking time and the positioning time could also affect the positioning error results. The bladder volume and BMI can also be further explored in future studies to investigate their effects on positioning errors.

Based on the findings of this study, future studies can further explore the application effect of dual positioning reference lines in radiotherapy of other tumors. In addition, advanced technologies such as SGRT can be combined to further optimize the setting of the positioning reference lines to improve the accuracy and reproducibility of positioning. Meanwhile, future studies can also explore the effects of dual positioning reference lines on the long‐term survival and quality of life of patients.

## 5 CONCLUSION

The application of dual positioning reference lines can significantly reduce the positioning errors and optimize the potential for refining the boundaries of the treatment target volume in VMAT for middle and advanced cervical cancer, particularly linear errors in the *Y* direction and rotational errors in the *RY* and *RZ*. This technique addresses the limitations of traditional single reference line positioning method, enhancing the reproducibility and stability of positioning, while maintaining treatment accuracy. It can also improve positioning efficiency, and thus provide clinicians with a simple, effective, and practical solution for precision radiotherapy, which is suitable for promotion and application in radiotherapy centers with limited resources.

## AUTHOR CONTRIBUTIONS

**Jinjin Feng**: Conceptualization; data curation; funding acquisition; investigation; methodology; writing—original draft; writing—review and editing. **Zaichun Shang**: Conceptualization; writing—original draft; writing—review and editing. **Binbin Ge**: Data curation; formal analysis; investigation; writing—review and editing. **Kaiyue Chu**: Data curation; investigation; writing—review and editing. **Jianhua Jin**: Data curation; formal analysis; investigation; writing—review and editing. **Jhingjing Shao**: Data curation; investigation; writing—review and editing. **Xinhua Zhang**: Conceptualization; investigation; methodology; project administration; writing—original draft.

## CONFLICT OF INTEREST STATEMENT

The authors declare that the research was conducted without any commercial or financial relationships that could potentially create a conflict of interest.

## ETHICS STATEMENT

This study was conducted in accordance with the Declaration of Helsinki and approved by the ethics committee of Nantong Tumor Hospital, The Affiliated Tumor Hospital of Nantong University (Approval No.2022–03). We obtained signed informed consent from the participants in this study.

## Data Availability

The original contributions presented in the study are included in the article/supplementary material. Further inquiries can be directed to the corresponding author.
